# Correction to “Self‐Assembly of Heterogeneous Ferritin Nanocages for Tumor Uptake and Penetration”

**DOI:** 10.1002/advs.75950

**Published:** 2026-06-01

Q. Liu, C. Wang, M. Zhu, J. Liu, Q. Duan, A. C. Midgley, R. Liu, B. Jiang, D. Kong, Q. Chen, J. Zhuang, X. Huang. *Adv. Sci*.2024, 11, e2309271.

https://doi.org/10.1002/advs.202309271


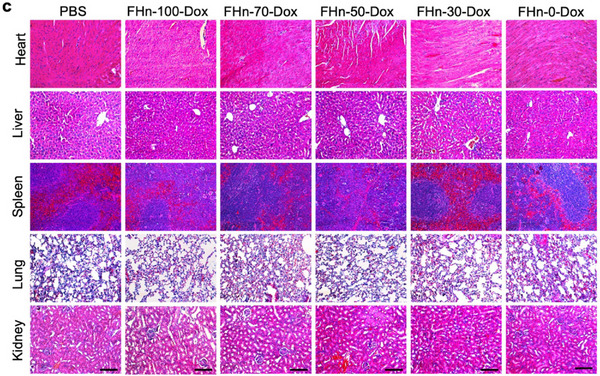


**Figure S7c** | In the originally published version of the article, the representative H&E staining image shown in the liver panel of the FHn‐50‐Dox group was incorrect. This error occurred during figure assembly and was inadvertently overlooked prior to publication. The correct representative image is provided below. Importantly, this correction does not affect the interpretation of the data or the conclusions of the study. We kindly request that Figure S7c be corrected as follows:

Corrected Figure S7c:


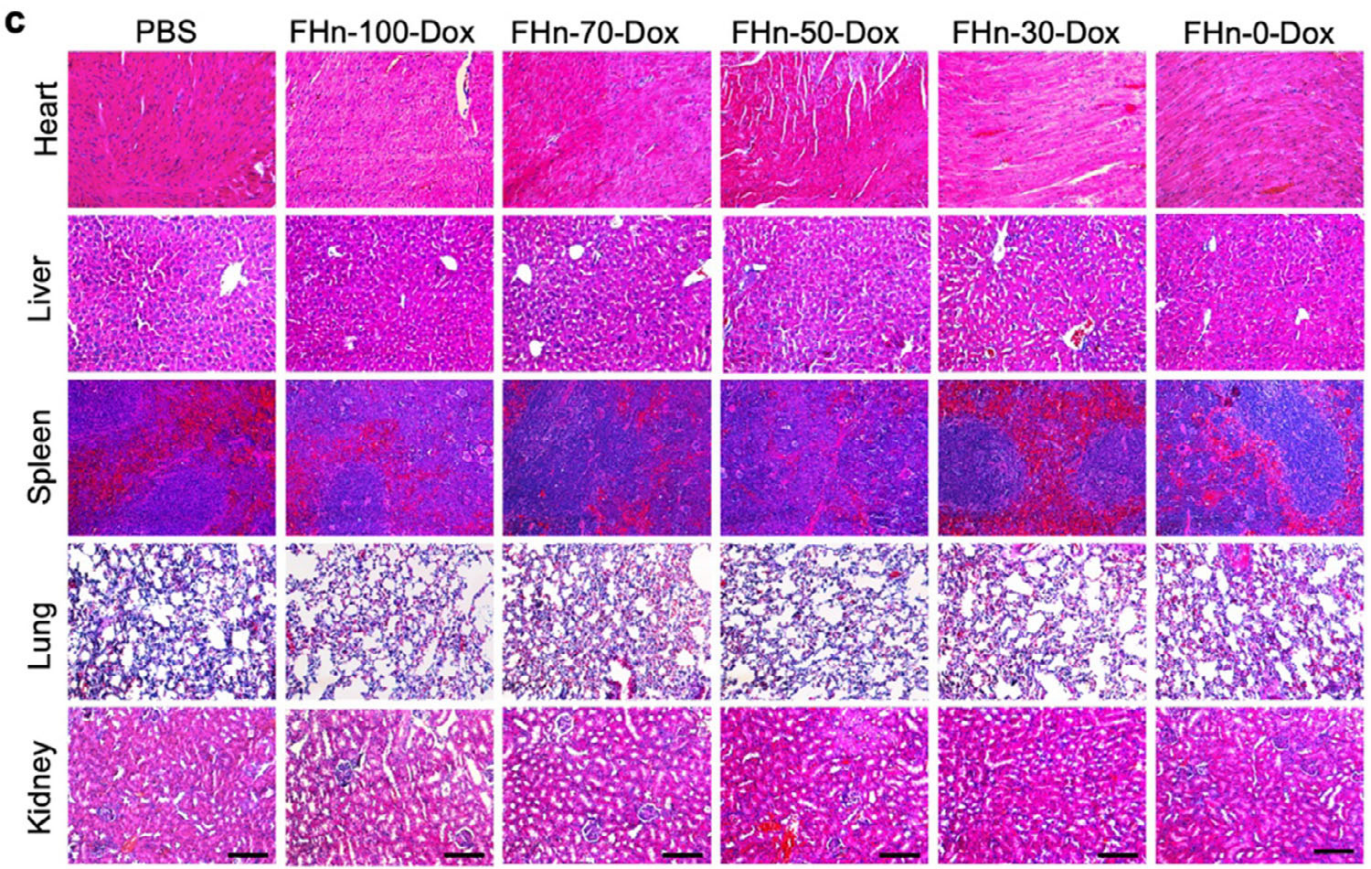


We sincerely apologize for this error.

